# Supplementary material for: Bacterial curli protein promotes the conversion of PAP248-286 into the amyloid SEVI: cross-seeding of dissimilar amyloid sequences
Source: PeerJ. 2013 Feb 12;1:e5. doi: 10.7717/peerj.5 (PMC3629062; doi:10.7717/peerj.5)
Supplement: Supplementary Information 1 [file peerj-01-5-s001.pdf]

Supporting Information

Bacterial Curli Protein Promotes the Conversion of PAP(248-286) into the Amyloid SEVI: Cross-Seeding of Dissimilar Amyloid Sequences

Kevin Hartman,[a,b] Jeffrey R. Brender,[a,b] Kazuaki Monde,[c,1] Akira Ono,[c] Margery L. Evans,[d] Nataliya Popovych, [a,b,2] Matthew R. Chapman,[d] Ayyalusamy Ramamoorthy [a,b]

[a] Departments of Chemistry, [b] Biophysics, and [d] Molecular, Cellular, and Developmental Biology, University of Michigan, Ann Arbor, MI 48109-1055, USA.

[c] Department of Microbiology and Immunology, University of Michigan Medical School, MI 48109-1055, USA..

[1] Present Address: Department of Medical Virology, Faculty of Life Sciences, Kumamoto University, Kumamoto 860-0811 Japan.

[2] Present Address: Genentech, 1 DNA Way South San Francisco, CA 94080.

Corresponding Author: Ayyalusamy Ramamoorthy, ramamoor@umich.edu, 930 North University, Ann Arbor, MI 48109-1055, USA.

CsgA
R1: S E L N I Y Q Y G G G N S A L A L Q T D A R N
R2: S D L T I T W H G G G N G A D V G Q – G S D D
R3: S S I D L T Q R G F G N S A T L D Q W N G K N
R4: S E M T V K Q F G G G N G A A V D Q – T A S N
R5: S S V N V T Q V G F G N N A T A H Q Y

CsgB
R1: Q A A I I G Q A G T N N S A Q L R Q G G S K
R2: L L A V V A Q E G S S N R A K I D Q T G D Y
R3: N L A Y I D Q A G S A N D A S I S Q G A Y G
R4: N T A M I I Q K G S G N K A N I T Q Y G T Q
R5: K T A I V V Q R Q S Q M A I R V T Q R

PAP$_{248\text{-}286}$
GIHKQKEKSRLQGGVLVNEILNHMKRATQIPSYKKLIMY

Figure S1. Amino acid sequences of CsgA, CsgB, and PAP$_{248\text{-}286}$. CsgA and B consist of five homologous subunits (R1-R5) that are believed to correspond to five β-sheets in the amyloid fiber.

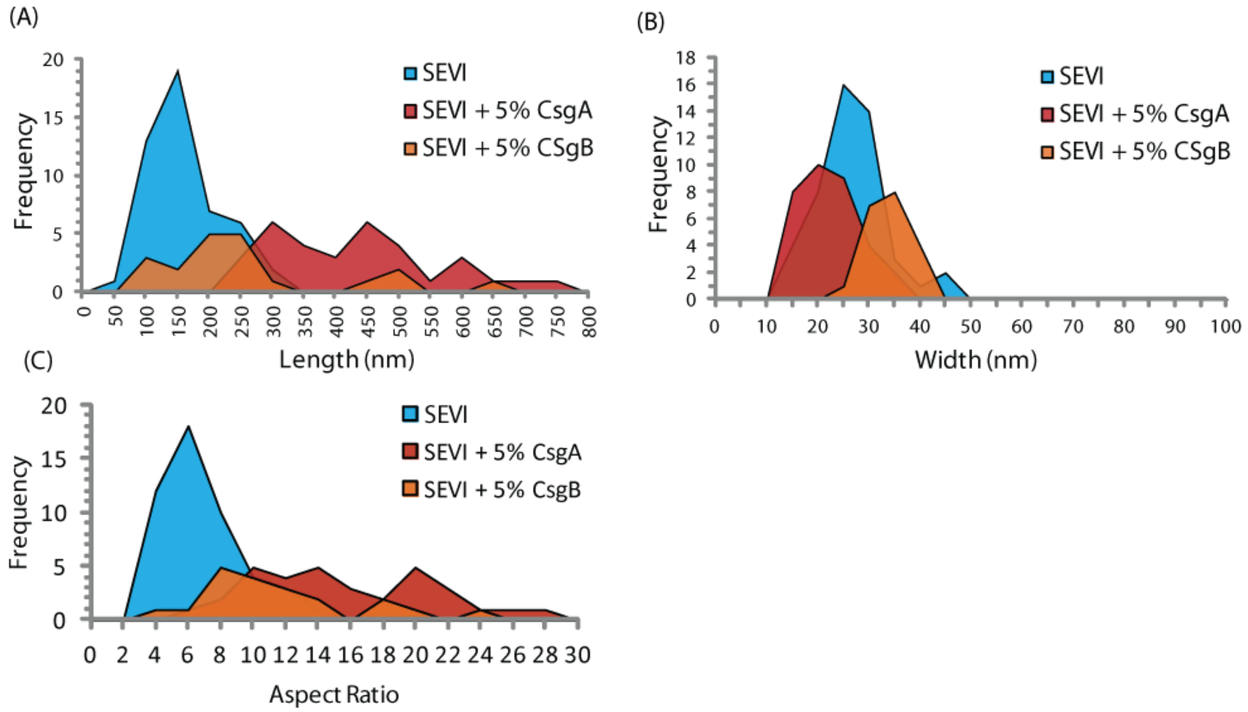

Figure S2 Distributions of fiber length (A), width (B), and aspect ratio (C) for seeded and unseeded samples as indicated.

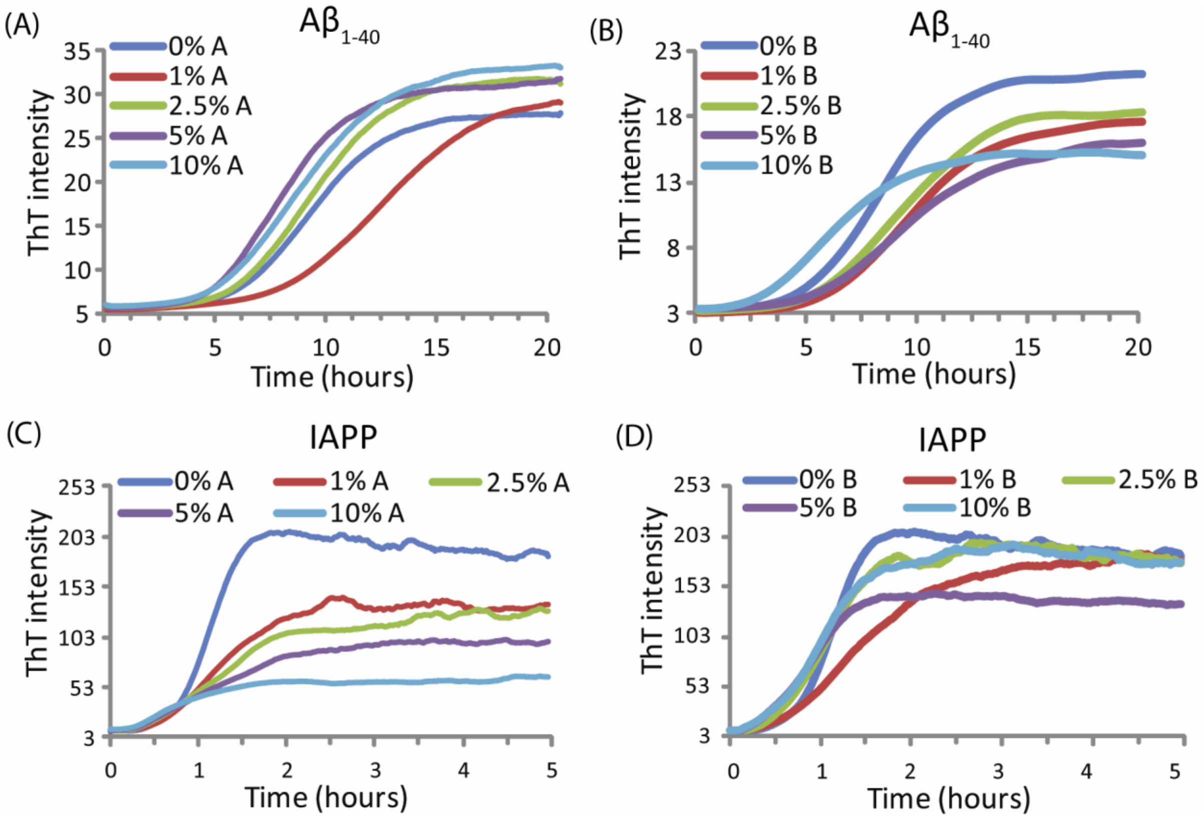

Figure S3. Kinetics of Aβ$_{1\text{-}40}$ and IAPP amyloid fiber formation in the presence of preformed fibers of CsgA and CsgB. ThT fluorescence measurements of Aβ1-40 **(Top)** and IAPP **(Bottom)** as a

function of CsgA **(left)** and CsgB **(right)** seeding concentration, expressed as a mole percentage of the Aβ_{1-40} and IAPP concentrations (5 μM and 2.5 μM respectively). Curves are averages for 3 measurements.

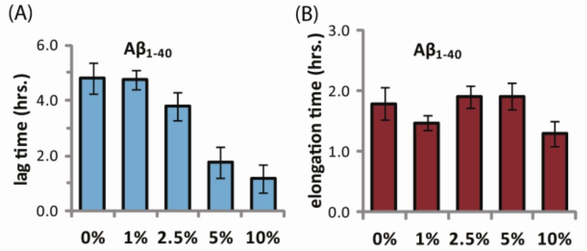

Figure S4. Seeding Aβ_{1-40} amyloid formation with preformed Aβ_{1-40} significantly affects the lag-time but has less impact on the elongation rate. Impact of preformed Aβ_{1-40} fibers on the lag time (A) and elongation time (B) of amyloid formation 5 μM Aβ_{1-40} (bottom) as molar percentage of the Aβ_{1-40} concentration.
